# Surgery

**Published:** 1857-05

**Authors:** 


### SURGERY.

1. INVERTED TOE-NAIL TREATED WITHOUT OPERATION. By *Robert Campbell*, M. D., Demonstrator of Anatomy in the Medical College of Georgia.—"Always dissatisfied with the various modifications of the operation that contemplates evulsion of the nail, which we have long deemed an unwarrantable measure of torture, in the treatment of this most *excruciating trifle*,—in the year 1851, having an opportunity of experimenting in a severe case of this affection, we endeavored to devise some expedient, which might answer the indication (of keeping the nail apart from the irritated flesh), by affecting the position and condition of the *flesh* itself, *by pressure*, as a substitute for the only plan we had ever seen proposed in the journals, or in the systematic works on surgery, whereby an operation was involved.

"From the entire success of this experiment, we have treated all the cases which have fallen in our way since, in the same manner—some of which were very unpromising in appearance—and have never had occasion to abandon it in any one case, or seek its modification. The condition of the part results most probably, from the use of too narrow a shoe, which contracts the toes into so small a compass, as necessarily to force the flesh at the margins of the great toe above the nail, from the lateral pressure of the inner side of the shoe, from within, and the other toes from without. The difficulty occurs most frequently, we believe, on the outer side of the great toe-nail, from the fact, that the second toe becomes depressed habitually, from this cause, and is impacted against the lower portion of that side of the great toe, and being pushed upward by the pressure of the ground from below, in walking, together with the lateral pressure, forces the flesh against the nail (which has not, necessarily, more than its normal breadth), until it becomes irritated, sore and swollen, the inflammation often resulting in a fungous growth, which is sometimes so sensitive as to disable the limb entirely.

"The indication is to adapt an apparatus of counter-pressure, at the same time training the second toe to such a position, that it will be available afterwards, as a natural, permanent compress, for the prevention of a repetition of such an obtrusion.

"This apparatus is formed of a bandage somewhat broader than the length of the nail—say $\frac{3}{4}$ths of an inch and $1\frac{1}{2}$ yards long, having a roll at one end about $\frac{1}{4}$th of an inch thick, to be used as a compress, to which the last turn of the bandage is tacked, to prevent the disposition otherwise to unroll on traction. This compress is applied in the groove, between the flesh (which is generally much enlarged) and the nail. The margin of the compress, at which the last turn of the bandage is sewed, must present always upward and opposite to the direction in which the pressure is to be exerted—i. e., if the outer border of the great toe on the right side is affected, the seam should be directed upwards and towards the left side of the patient. Thus applied, the bandage will become somewhat wound around the outer side of the compress, which is to be pressed, at first, very gradually and tenderly, but somewhat forcibly, downwards and outwards, until secured in that position by several turns of the bandage around the great toe, carrying it first down, between that member and the second toe, and around several times. The great toe is then to be depressed and forced outwardly, under the second, which is placed upon the compress, and lightly bound in that position by a few turns of the bandage.

"The advantages of this treatment over the operation, will, no doubt be apparent to every one—as substituting a practice as shocking to the operator as agonizing to the subject; which considerations, in many instances, rendered the art a perfect nonentity in reference to these cases—deterring the surgeon, as well as the patient, from all attempts at relief, and entailing upon the latter months, perhaps years, of comparative indolence or of unmitigated suffering, and upon the former, the opprobrium of incompetency on account of the frightful nature of the only alternative.

"This bandage should be reapplied, at least as often as once a week. If the prominence of flesh, against which the compress is to rest, is very irritable, a layer of lint, anointed with simple cerate, should intervene. At the first impression of the compress on the sore flesh, the patient experiences some degree of pain or soreness; but the very prompt abatement of suffering following the relief of the inflamed tissues from the irritation of the sharp nail, and also the diminished turgescence of the part, induced by the uniform pressure of the compress and bandage, will prove an ample recompense. The comfort of the patient and celerity of recovery are much enhanced by keeping the limb in an elevated position. It is a matter of surprise, bordering upon astonishment, to see the great diminution of sensitiveness in the part, at each succeeding application of the apparatus. Six or eight weeks is time sufficient for relief, generally, or for such an improvement as to render it safe to intrust the case to the care of the patient.

"We have been thus minute and comprehensive in the above detail, in order that we might be comprehensible—hoping that the suggestions which, confiding in our experience, we have ventured to advance—though contrary to "written law," may not fall into disrepute through inefficiency of application."—*Southern Med. & Surg. Journal*, Feb. 1857.

2. LAGOPHTHALMOS, FROM PARALYSIS OF THE ORBICULARIS PALPEBRARUM. By *George C. Blackman*, M.D., Professor of Surgery, Medical College of Ohio.—"John Plunket, æt. 28, was admitted into the Commercial Hospital, Oct. 27, 1856. In March of the same year, he contracted two chancres on the glans penis, which were cauterized by a practitioner, and healed in the course of a fortnight. A bubo followed on the left side, which attained considerable size, but never suppurated. This, in a few weeks, disappeared, and he afterwards suffered from pains in his bones, and became covered with a pustular eruption. He had been of intemperate habits, and had had several attacks of delirium tremens, one of which occurred after contracting the syphilitic affection, but before he entered the hospital. During these attacks he suffered severely from pain in his head. At the time of his admission, he was unable to close his left eyelid, and the distortion of that side of the face left no doubt as to the existence of paralysis of the *portio dura* nerve. The deathlike appearance of the affected side contrasted strangely with the other when the latter was called into action, owing to the wrinkled forehead as far as the median line, and the dragging of the nose and mouth toward the unpalsied side. In his efforts to whistle, the orbicularis oris would contract on the right, but not at all on the left side, and in speaking, the cheek would 'flap to and fro,' from the paralysis of the buccinator muscle. This complication, so generally observed in these cases, is difficult of explanation, as the buccinator is supposed to derive its chief motor power from the trifacial nerve. In masticating his food, our patient was compelled to employ his fingers to prevent the accumulation of food between the teeth and the cheek. There was severe pain in the trunk of the *portio dura* just after it emerges from the stylomastoid foramen, and this was undoubtedly due, as well as the paralysis of the muscles of the face, to the pressure of an exostosis from the skull, just beneath the mastoid process. On first examination this was regarded as an indurated gland, but subsequent more careful investigation established the existence of a periosteal deposition and induration, or, an exostosis. It should be stated, that the sensibility of the cheek was slightly impaired.

"Before his admission he had been under various kinds of treatment. Mercury had been administered, and blisters had been applied behind the ear and upon the nape of the neck. The latter, the patient seemed to think, had but aggravated his sufferings. As he had recovered from the effects of his intemperance—as far as his general health was concerned,—I directed a cathartic, after the operation of which, he was to take pills of the protoiodide of mercury, one grain each, three times per day. These were given for several days, and then the iodide of potassium was substituted—ten grains three times a day. In the course of a week the dose was increased to fifteen grains three times a day. For something like a fortnight, but little impression seemed to be produced, and I determined, if the affection continued thus obstinate, to resort to Dieffenbach's operation of dividing the levator palpebræ. In the early part of December, however, the power of the orbicularis palpebrarum was decidedly increased, and the other symptoms of paralysis began to subside. About the 20th of the month, he could completely close the eyelids, and at the present time, January 18, but very slight deformity of the face remains. The exostosis or periosteal induration, has materially diminished, and the improvement of the patient has kept pace with this diminution.

"To those acquainted with the experiments of Sir Chas. Bell upon the *portio dura* nerve, in 1821, and those repeated by Mr. Shaw, MM. Mayo, Eschricht, Magendie, and Conget, the above case cannot be devoid of interest. The coexistence of impaired sensibility on the same side of the face, may be regarded by some as an evidence that the trifacial nerve was also involved, and that the paralysis of the *portio dura* was dependent on intracranial disease. While it is universally admitted that the *portio dura* is the *general motor* nerve of the face, it is by no means established that it does not possess sensory endowments, or that the latter depend altogether upon anastomoses with other nerves, after its escape from the stylo-mastoid foramen. It is probable, however, that the *greater* part of its sensibility is derived from the branches which it receives from the trifacial; yet M. Sappey, in his *Traité d'Anatomie Descriptive*, states that it receives a small branch from the posterior surface of the spinal cord, from which sensitive portions of nerves take their origin, and that, with Bischoff, Bartold, Ch. Robin, and others, we must admit that the *portio dura* is also a nerve of sensation.

"Dr. J. H. Bennet, in his work on *Cancerous and Cancroid Growths*, relates (p. 83), a case in which a small indurated swelling, below the left ear, gave rise to paralysis of that side of the face, attended with lagophthalmos. This tumor proved to be cancroid affection of the parotid gland, which destroyed the patient.

"Professor Graham, of the Medical College of Ohio, informs us that he has seen a patient cured of a paralysis of the *portio dura*, by the extirpation of a morbid growth which pressed upon it, between the mastoid process and the ramus of the jaw. Mr. Mackenzie, in his Treatise on the Eye, remarks that the disease has been known to arise from the pressure of a lymphatic gland lying between the jaw and the mastoid process. But the case we have given in detail is the only one with which we are acquainted where the morbid growth producing the paralysis was of a syphilitic character. Vidal and other writers on syphilis, refer to cases of amaurosis and various cerebral affections due to this source, and to paralytic affections of the lower extremities dependent upon similar growths, from the spine encroaching upon the spinal cord, and cured by the iodide of potassium. In conclusion, we would refer those interested in the study of 'Bell's

Paralysis of the Face,' to Dr. Todd's 'Clinical Lectures on the Nervous System,' a work which, upon this subject, we believe to be unequalled in the English language.—*West. Lancet,* February, 1857.

3. SPINA BIFIDA—THREE CASES FROM ONE MOTHER. By *E. M. Pendleton,* M.D., of Sparta, Georgia.—"In a practice of twenty years, I have seen but four cases of that terrible congenital disease, or malformation, termed Spina Bifida. The first, was in consultation with Dr. Lynch, of Warren County, which terminated fatally, at several years of age. The sac, containing water and connected with the brain through the spinal column, was at the end of the os coccygis. The other three cases have occurred within the last five years, and singularly enough, were all the offsprings of the same mother, a lady of this county, of the first respectability. Her first child, a son, had a very small sac on the left side of the spinal column, in the lumbar region, which did not seem to connect specially with the spine, and but for the further developments of the case, would not have produced much apprehension. Hydrocephalic symptoms, however, supervened in a few months, the head became enlarged and the child had occasional spasms; it died in a short time.

"The second child was apparently healthy; but as the parents had removed from their former residence, I lost sight of the case, until summoned to attend it, in consultation with another physician. It labored under the effects of catarrh and dentition, the brain suffering greatly. It died in a few days after I saw it.

"In the third case, I attended the accouchement, and found, as soon as the child was born, that it presented symptoms of paralysis. Upon examination, the same fearful sac, at about the same point of the lumbar vertebræ, presented itself, only it was much larger than the other; the legs crossed, and were perfectly paralyzed. The head was larger than natural, and soon exhibited hydrocephalic symptoms. It lived several months, and cried nearly all the time of its waking existence.

"Several months since, I was summoned to the same lady in her fourth confinement. I went with fear and trembling, feeling a strange premonition that I would have the same difficulty to contend with as formerly. Anxiety and apprehension were depicted on the countenances of both parents, and I could but offer a silent prayer that this one might be well formed. But, alas! as the child presented itself to our vision the first time, we detected the same inability to move its lower extremities, the same malposition of the legs, and the same fearful fissure in the back, only larger, apparently, than either of the others. We turned away sickened at the sight. In other respects, the child seemed to be healthy and sprightly. It, too, soon followed its predecessors to the grave.

"What can be the cause of this singular concatenation of unfortunate births, from parents who are both well formed, vigorous, and healthy, in every possible respect? The father is of a bilious, lymphatic temperament; the mother, sanguineo-nervous—the parents of both still alive and healthy. This would seem to be one of the best crosses imaginable. The maternal grandmother, however, springs from a family of over sanguineous temperaments and scrofulous diatheses. They are very prone also to intermarriages. Her immediate offspring, however, are all healthly, and I cannot conceive that there exists any just cause of suspicion on this ground.

"The case is one of great interest to those who love to investigate the profound intricacies of the physiological and pathological relations of parents and offspring. We feel an especial interest in presenting these cases, hoping that such untoward effects may be traced to their legitimate causes, and some prophylaxis be instituted, that may arrest the calamity in future; if not in the case under consideration, at least in some which lie in the lap of the future."—*Southern Med. & Surg. Journal*, April, 1857.

4. REMOVAL OF TUMORS BY A NEW METHOD.—"Dr. Simpson, of Edinburgh, has been experimenting on the removal of tumors by a method novel in this country. He introduces a hollow acupuncture-needle, or very fine trocar, into their tissue, and injects a few drops of some irritant liquid—such as a solution of chloride of zinc, perchloride of iron, or creasote. The effect has been to destroy the vitality of the tumors so treated, and they have been separated by a process of enucleation. We have seen a somewhat similar plan adopted in Paris by M. Maisonneuve. He has slender stylets made of a paste composed of flour, water, and chloride of zinc. These are baked. A puncture is made in the tumor, the caustic style is inserted, broken off, and left. We saw several malignant tumors treated in this manner, and some cases in which a healthy granulating surface was left after the separation of tumors which had been destroyed in this manner."—*Times and Gazette, Feb.* 7, 1857.

5. NEW MODE OF EMPLOYING THE TAXIS IN HERNIA. *By B. G. Darley, M.D.*—"I have read in your last number a report of a lecture on the taxis in hernia by Mr. Skey. I am induced to send you a mode of manipulation which he has not mentioned, nor any one else that I am aware of, and which I found very successful in a case I was called to see some time since; it may be of use to him as well as to others. It was an inguinal hernia, in a young man, that had been strangulated for the greater part of a day; size not very large, but very tense, and hard, attended with the usual symptoms,—vomiting, confined bowels, &c. I tried the taxis, as it is called, but failed. I then made him bend both his knees, and took one under each arm and with his head hanging, I gave a good shaking as long I could hold him, telling him to press up the tumor as well as he could himself. On letting him down, I found the tumor not so low down in the scrotum as it was; it was soft, and holding it in my hand for a minute it went up. The *rationale* of this proceeding is very easily explained, and is on the same principle which succeeded in dislodging the half-sovereign from the larynx of Mr. Brunel, when the surgeons were about to open his wind-pipe."—*Dublin Med. Press, Feb.* 4, 1857.

6. IODINE INJECTIONS IN THE TREATMENT OF OVARIAN DROPSY.—In a late discussion before the Medico-Chirurgical Society of Edinburgh, Professor Simpson stated, that "He had now employed these injections in twenty or thirty cases, with varying results. In the first operation, the first, he supposed, in which it had been used in Great Britain, the tumor is still present, but never has again increased to any great size. Sometimes the injection in his hands had proved quite successful. Lately, he saw two patients on whom he had operated three years ago. In one of these cases, a young person of twenty or twenty-two, who had been once or twice tapped before, the dropsical tumor was of very great size, and the patient's health and strength were rapidly breaking down when the iodine injection was had recourse to. There has been no return of the dropsy, and the patient is now quite well and strong. He lately saw an elderly patient, upon whom he had operated about the same time, with a similar successful and apparently permanent result. In other cases, the iodine injection had been completely or partially successful—partially in several, inasmuch as it had obliterated the largest cyst in the multilocular tumor, but had not prevented the remaining smaller cysts from growing and developing. In some, on the other hand, it had so far entirely failed, that the cyst, operated on and injected, had again refilled; but, perhaps, as a general rule, not so rapidly as when no injection was used. The failures were, in special instances, perhaps traceable to the iodine being too much diluted by the fluid left in the cyst; to the quantity of iodine used being too small, or too weak; to care not being taken to bring it in contact with the whole interior of the cyst, and other possibly avoidable causes. No doubt it was a valuable means in some cases, especially where the dropsy was principally limited to one or two large cysts: and no doubt it would fail in others, especially where the tumor had several large cysts developing simultaneously. The iodine injection was seldom attended with much pain, or with any severe local or constitutional irritation. Out of the twenty or thirty cases in which he had injected ovarian cysts with iodine, in only one instance had death subsequently occurred, namely, in a patient to whom he was called by Dr. Monroe, of Dundee. The dropsical distension of the abdomen in this patient was, before tapping, greater, he believed, than he had ever before witnessed, and the iodine injection was used at the first tapping. Was the fatal result attributable to the tapping or the injection? He had now used the iodine injection so often, without any marked local suffering or constitutional reaction, that he was inclined to doubt if the iodine were in any degree blamable; while he he had so frequently seen danger and death follow first tappings, and where nothing but tapping was used, that he believed the result was to be ascribed to the paracentesis, and not to the injection. Dr. Duncan spoke of tapping being said to be fatal in the proportion of 1 to 4. He had never seen such a high general mortality ascribed to ovarian paracentesis; but Mr. Southam had published a limited table of cases, in which the operation was fatal in first tappings, in the proportion, if he remembered aright, of 1 in 5. And when Dr. Duncan spoke of tapping being a safe operation, because it was occasionally repeated 40 or 50 times upon the same patient, he entirely forgot, he feared, the all-important fact, that the danger of ovarian tappings attached principally to the *first* operation, and comparatively little to the subsequent repetitions of the paracentesis. During the last few years, since using iodine injections, he had seen, in hospital and consultation practice, several patients die after ovarian tapping, in two or three of whom iodine was intended to be used, but was, for the sake of the treatment, for various causes, fortunately omitted. In one case, where the iodine apparatus went wrong when about to use it, he successfully adopted a plan of practice which he had suggested in writing, namely, he had established a fistulous opening between the secreting cavity of the cyst and the absorbing cavity of the peritoneum, by compressing and bursting from day to day, the ovarian collection through the trocar-wound in the walls of the ovarian cyst. A wound made by a four-sided trocar would be more easily changed into such a fistula than a wound with the usual triangular-shaped instrument. In the few cases of ovarian dropsy cured by nature and accident, the establishment of such a fistulous opening was the means employed. Other measures, he had reason to hope, might yet be happily devised for the treatment of this very grave disease, seeing the attention of the profession was so strongly called to it at present. As to the operation of complete extirpation, he still held the same views which he had expressed to the society several years ago, namely, that there were some cases of ovarian dropsy in which other milder measures having failed, as being inadmissible, ovariotomy was justifiable on the same principles and grounds as most of the other major operations in surgery for chronic incurable disease were justifiable."—*Edinburgh Med. Journal*, Feb. 1857.

7. SENILE GANGRENE. At a late meeting of the New York Pathological Society, "Dr. Markoe exhibited the aorta, common iliac, femoral, and popliteal arteries, taken from a patient dying from senile gangrene. The patient was an old man, between 60 and 70 years of age; while at sea, a passenger stepped on his great toe, on which he had long had a bunion; inflammation followed, and the surgeon on shipboard applied some nitrate of silver, which seemed to aggravate the symptoms, and shortly after a sloughy appearance took place round the original bruise. Dry gangrene spread gradually from this point on to the great toe, reaching to its base and gradually involving more or less of the smaller toes, spreading up half-way along the dorsal surface of the metatarsus. This was accompanied by a good deal of irritative fever. The gangrene spread slowly after his admission to the hospital, but three or four days before the operation it presented an appearanee which he has since regarded as of more significance than was at the time attributed to it. This appearance was that of a tongue or peninsula of reddened and inflamed integument, running up on the inner aspect of the foot above the ankle, and gradually falling into mortification. This seemed more like the mode of progress which obtains in mortification from obstructed arteries. Dr. Markoe had never seen this mode of rapid irregular extension in ordinary senile gangrene from enfeebled capillaries. The operation was performed below the knee, and, contrary to our previous experience, the mortification attacked the stump, the irritative fever increased, and the patient died within a week after the operation. He had examined the case after death with a great deal of interest, and the specimens presented seem to explain the unfortunate rasult.

"*Autopsy.*—The heart is nearly normal, presenting only some slight atheromatous deposits around the valves. In the aorta are seen numerous patches of atheroma; some of the plates are calcified, others have disappeared, leaving a denuded surface. All the smaller arteries down to where the tibial was cut in the amputation are extensively calcified. This is still more marked on the tibials and radials, etc., some of which seem to be mere bony tubes or cords. Besides this calcification of the coats, however, there is observed all along the great vessels, and more noticeably in the smaller trunks, a puckering and irregular contraction of the coats of the vessels sufficient to diminish their calibre at different points. At the lower part of the femoral artery an annular contraction exists, so considerable, that the calibre of the tube is reduced to one-third of its proper dimensions. This seems to be due to a puckering-in of all the coats of the vessel, at the point of ligature of the popliteal; although the artery is extensively diseased, yet a clot has formed within, and everything about the ligature seems to promise a perfect and safe closure of the arteries. We have here, therefore, not only the enfeebled condition of the capillaries, which is the ordinary cause of senile gangrene, but we have also an obstructed condition of the trunks of the vessels, just such, in effect, as obtains when these trunks are obliterated by adhesive inflammation. This, as far as Dr. Markoe's experience went, was a rare combination, and determined, no doubt, the spreading mortification, and the fatal termination of the case. If we have nothing to deal with but the enfeebled capillaries of old age, by amputating higher up, nearer the centre of circulation, and therefore through stronger capillaries, we have no return of mortification in the stump, and, if other circumstances are favorable, the patient may do well. Every surgeon knows, on the other hand, the danger in amputating too soon in those cases, in which, in young people, gangrene is the result of arteritis with obstruction. Dr. Markoe believed that, as the pathological condition was so different in the two cases, a clinical distinction might be drawn; a distinction, if correctly drawn, of great practical importance, in reference to the question of amputation.

"Dr. McCready stated that he had met with a case where dry gangrene in three fingers was caused by a tumor pressing upon the subclavian, in a woman, 30 years of age. In another case of disease in the femoral and obliteration of the anterior tibial arteries, there was dry gangrene of the toe.

"In reply to a question from Dr. Clark, Dr. Markoe said, that he did not mean to draw a distinction between inflammation and obstruction of arteries, for that in their results, as far as producing gangrene was concerned, they were nearly identical. The distinction he was insisting upon, was one between gangrene produced by loss of power in the distant capillaries, as of the feet and hands of old people, and gangrene produced by obstruction of principal trunks, such as occurs in young persons from arteritis. In the affection of the capillaries, Dr. Markoe had never seen the disease spread beyond the ankle, while in obstructed arteries a whole limb often perishes, usually by successive attacks of gangrene. In the first case we may amputate safely; in the second, we operate with doubt, hesitation, and danger, because we do not know how high up the obstruction may be.

"Dr. E. R. Peaslee remarked, that he had never yet known of a case of gangrene in a young person, where it was not owing to some obstruction; but, on the other hand, it seemed to him that the treatment of gangrene in old persons, went to show that it also often depended upon obstruction in the larger arteries. Practically he thought it might be difficult to decide what was best to do, for if amputation be resorted to, gangrene is apt to set in.

"Dr. James R. Wood thought this to be a very interesting subject, especially in a medico-legal point of view. He stated that the fact was settled long ago, that disease of the capillaries of the extremities was a cause of gangrene in both young and old. Also, that gangrene was sometimes the result of obstruction. He thought that the diagnosis between these two forms ought to attract the attention of the society, and hoped that either the subject would be discussed at some future day, or be referred to a committee to report."—*N. Y. Journal of Medicine*, March, 1857.

